# First study of hepatitis delta virus in Algeria: Seroprevalence and risk factors in Setif region (east of Algeria)

**DOI:** 10.4102/sajid.v34i1.110

**Published:** 2019-10-23

**Authors:** Abdelkader Gasmi, Wahiba Guenifi, Amel Ouyahia, Mounira Rais, Houda Boukhrissa, Abderahmen Hachani, Salah Mechakra, Slimen Laouamri, Abderezak Touabti, Abdelmadjid Lacheheb

**Affiliations:** 1 Division of Infectious Diseases Teaching Hospital, Faculty of Medicine, University Ferhat Abbes Setif, Setif, Algeria; 2 Division of Epidemiology Teaching Hospital, Faculty of Medicine, University Ferhat Abbes Setif, Setif, Algeria; 3 Division of Microbiology Teaching Hospital, Faculty of Medicine, University Ferhat Abbes Setif, Setif, Algeria

**Keywords:** Hepatitis Delta Virus, prevalence, risk factors, Setif, Algeria

## Abstract

**Background:**

No recent data are available on hepatitis delta virus (HDV) prevalence in Algeria. For this reason we conducted an epidemiological study, cross-sectional seroprevalence of HDV in the region of Setif.

**Methods:**

Between 2011 and 2014, sera samples of 500 patients (carrying HBsAg) admitted to the Division of Infectious Diseases Teaching Hospital, Setif (east of Algeria), were tested for anti-HDV-IgG ab (ETI-AB-DeltaK-2).

**Results:**

The prevalence of HDV obtained is estimated at 2.4%. The prevalence ranges from 1% in chronic hepatitis to 11.1% in cirrhotic hepatitis (low endemic area). Seropositivity rate is closely correlated with age (Odds ratio [OR] = 9.98, *p* = 0.000) and gender (OR = 0.24, *p* = 0.025); it reaches 58.3% in the age group of 51–60 years and 0% in children (age group 1–15 years); it represents 75% in females and 25% in males. The presence of familial cases of HBsAg positive (OR = 4.54, *p* = 0.006), the endoscopic procedure (OR = 6.54, *p* = 0.000) and tattooing (OR = 20, *p* = 0.000) were found to be the transmission risk factors. A statistically significant relationship was found between the positivity of anti-HDV and advanced liver disease, cirrhosis (OR = 9. 16, *p* = 0.000). A significant correlation was found between the positivity of anti-HDV with diabetes (OR = 6.83, *p* = 0.000), obesity (OR = 4.19, *p* = 0.009) and viral suppression B (OR = 5.69, *p* = 0.003).

**Conclusion:**

Our results show that HDV infection is low in Algeria. Research for total anti-HDV should be part of the initial assessment of patient care with viral hepatitis B as well as the prevalence of other viruses (hepatitis C [HCV] and HIV). A multicentre study should be carried out to know the importance of HDV infection and identify the risk groups.

## Introduction

First described in 1977 by Rizzetto et al.,^1^ the hepatitis delta virus (HDV) is a small virus 1.7 kb RNA, single-stranded, negative polarity considered a human agent. Biological characteristics not fully completing the virus definition criteria and its dependence on a helper virus^2^ has placed it under the satellite virus group.^3^ In 1993, the International Committee on Taxonomy of Viruses proposed to classify it in a member of the free-floating genus Deltavirus^4^ of which it is the sole representative.^5^ One of its characteristics is its high genetic variability with eight separate genotypes HDV (HDV1–8).^6,7^ Hepatitis delta is an ubiquitous transmissible infection, reported in every country in which it was sought.^8,9^ Nevertheless, there is a varying prevalence from one country to another country and from one region to another region within the same country. Early studies in the eighties have found a mean prevalence estimated at 5% in the carrier population of HBsAg.

Hepatitis delta infection remains a major public health problem, and it is currently estimated that, worldwide, between 15 and 20 million people are positive for the viral hepatitis delta (VHD) and it affects all ages, but its distribution is not uniform.^10,11^ The epidemiology of HDV has changed; in fact, factors such as vaccination and public health measures against acquired immunodeficiency syndrome (AIDS) combined with improvements in hygienic conditions have contributed to control the hepatitis B virus infection and, as a direct consequence, the decrease in the prevalence of infection with HDV.^12,13^

Algeria is exposed to the risk of reintroduction by migrants, that is, neighbours, coming mainly from its southern countries: 29% in Niger,^14^ 19.7% in Mauritania^15^ and 13.9% in Mali.^16^ Furthermore, we noted the presence of new genotypes such as the VHD5 genotype that represents 10.7% of isolated strains in Mauritania.^7^

**FIGURE 1:**
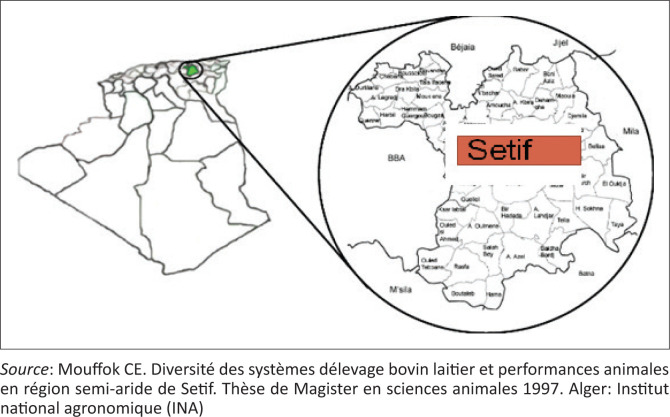
Location of Setif city.^17^

In Algeria, the epidemiology of this hepatitis is still very little known. Only four studies were conducted on limited numbers and populations. For this reason, we conducted a cross-sectional seroprevalence study of HDV in the Setif region (Figure 1).^17^

## Materials and methods

Between 2011 and 2014, sera samples of 500 patients (carrying HBsAg) admitted to the Division of Infectious Diseases Teaching Hospital, Setif (east of Algeria), were tested for anti-HDV-IgG ab (ETI-AB-DeltaK-2). The patients are from five cities in the east of Algeria (Setif, Bourdj-Bou-Arrerdj, Msila, Mila and Bejaia).

**Inclusion criteria:** All HBsAg patients in this study at the Division of Infectious Diseases Teaching Hospital, Setif.

**Exclusion criteria:** Patients refusing to participate.

A questionnaire was completed for each patient including information about age, sex, marital status, number of wives and children, socio-economic level, risk exposure during life, discovery circumstances, comorbidity and specific situations such as, overweight and obesity, diabetes, pregnancy, history of hemodialysis and co-infection with HCV and co-infection with HIV.

Results in clinical and biochemical examinations data were correlated with those of the HBV viral load. According to the stage of their liver disease, patients were divided into four groups: first group, acute hepatitis B; second group, chronic hepatitis B; third group, cirrhosis; and fourth group, hepatocellular carcinoma.

## Statistical analysis

Data analysis was performed using SPSS version 21.0 (SPSS Inc., USA). We used the technique of descriptive statistics with the estimated prevalence with a confidence interval (CI) ww 95%; chi-square test and Fishers exact test were used for the comparison of distributions and calculation of measures of epidemiological associations (odds ratio [OR]) with 95% CI. A p-value < 0.05 was considered significant.

### Ethical consideration

The study was approved by the research ethical committee of University Ferhat Abbes Setif, Algeria. All participants gave their consent before data and blood samples were collected.

## Results

The study of 500 sera samples from patients with HBsAg in different stages of the disease shows 12 patients having anti-delta positive total IgG, a prevalence equal to 2.4% with a 95% CI [1.1% – 3.7%].

The prevalence of HDV is higher among women (75% of positive anti-HDV). The difference was statistically significant (OR = 0.24, *p* = 0.025) (Table 1).

**TABLE 1:** Relationship with demographic characteristics, diabetes, pregnancy, history of hemodialysis, co-infection with HCV or HIV, biological and virological parameters and hepatitis delta virus.

Variable	IgG-anti-delta positivity		IgG-anti-delta negativity		*P*	OR	OR: Min-Max
	Positive	%	Negative	%			
**Age**							
Mean of age	55.5 ± 12.5	-	38.5 ± 15.5	-	0.000	-	-
Age group (51–60)	7	58.3	10	83.3	0.000	10.0	3–32.5
**Gender**							
Male	3	25	281	57.6	0.025	5.0	0.06–0.9
Female	9	75	207	42.4	-	-	-
**Marital status**							
Single	0	0	132	27.9	0.03	1.03	1.01–1.05
Married	12	100	341	71.1	-	-	-
**Number of children**							
Means ± SD	4.83 ± 2.65	-	2.83 ± 2.31	-	0.004	-	-
≥ 5	7	58.3	68	19.9	0.001	5.6	1.7–18.2
**Profession**							
Unemployed	10	83.3	259	53.1	0.03	4.4	0.9–20.3
Officials	1	8.3	68	18	0.38	-	-
Liberal officials	1	8.3	85	17.4	0.41	-	-
Students	0	0	43	8.8	0.28	-	-
Health workers	0	0	13	2.7	0.32	-	-
**BMI (Kg/m^2^)**							
Means ± SD							
Overweight (BMI: 25.00–29.99)	2	16.7	173	36.6	0.15	0.3	0.1–1.6
Obesity (BMI ≥ 30)	6	50.5	91	19.2	0.009	4.1	1.3–13.3
Diabetes	5	10.6	46	9.4	0.000	6.9	2–22
Pregnancy	0	0	31	15	0.20	-	-
History of hemodialysis	0	0	12	2.6	0.58	-	-
Co-infection with HCV	1	8.3	26	5.3	0.64	-	-
Co-infection with HIV	0	0	3	0.6	0.87	-	-
**ALT (IU/L)**							
Means ± SD	52.6 ± 19	-	45 ± 44.7	-	0.62	-	-
> 40	8	80	159	36.6	0.005	6.9	7.3–160.4
**Platelets** (*n*/**mm**^3^ × 10^6^)							
Means ± SD	172 ± 89	-	200 ± 81	-	0.23	-	-
< 150 000	7	58.3	352	72.1	0.29	34.3	7.3–160.4
HBeAg positivity	1	33.3	72	26.6	0.79	-	-
**Serum HBV DNA**							
< 100 IU/mL	5	50	65	14.9	0.003	5.7	1.6–20.2
> 4.3 log (10) copies/mL	2	20	102	23.4	0.78	-	-

ALT, alanine transaminase; PLT, platelets; SD, standard deviation; OR, odds ratio; Min, minimum; Max, maximum.

The average age of patients with VHD (55.5 ± 12.5 years) is significantly higher than that of patients infected with HBV alone (38.56 ± 15.5 years), and the difference was statistically significant (*p* = 0.000).

The city of Msila displays the highest rate of 6.9% 0% – 17.2%, followed by the city of Bourdj-Bou-Arrerdj with a rate of 2.9% 0% – 7.2% and the city of Setif with a rate of 2% 0.8% – 3.5%. In the city of Bejaia and Mila, the prevalence rate is zero (Table 2).

**TABLE 2:** Prevalence of HDV by city.

City	*N*	%	IgG-anti-delta+		*P*
			Positive	%	
Setif	394	78.8	8	2.0	0.29
Bourdj-Bou-Arrerdj	69	13.8	2	2.9	0.77
Msila	29	5.8	2	6.9	0.10
Mila	4	0.8	0	0.0	0.75
Bejaia	4	0.8	0	0.0	0.75

**Total**	**500**	**100**	**12**	**100**	**-**

Statistical analysis revealed a significant relationship between marital status and positivity of anti-HDV (OR = 1.03, *p* = 0.03), but there was no relationship between the number of wives and positivity of anti-HDV. The average number of children in patients with VHD (4.83 ± 2.65) is significantly higher than that of those infected with HBV alone (2.83 ± 2.31), and the difference is statistically significant (OR = 1.03, *p* = 0.004). A significant relationship was found between certain risk factors and transmission (the positive family history of hepatitis B OR = 4.54, *p* = 0.006, endoscopy OR = 6.54, *p* = 0.000, tattoos OR = 20, *p* = 0.000) (Tables 1 and 3).

**TABLE 3:** Relationship with risk factors and hepatitis delta virus.

Variable	IgG-anti-delta positivity		IgG-anti-delta negativity		*P*	OR	χ^2^ test
	Positive	%	Negative	%			
Dental procedures	10	83.3	397	81.4	0.86	1.1	0.03
Blood transfusion	3	25	123	25.2	0.98	0.9	0.20
Surgery	8	66.7	235	48.2	0.20	0.9	1.6
Endoscopy	7	58.3	86	17.6	0.000	6.5	12.8
Sexual	3	58.3	62	12.7	0.21	2.2	1.56
Cupping	2	16.7	53	10.9	0.52	1.6	0.40
Positive family History	7	58.3	115	23.6	0.006	4.5	7.67
blood exposure accident	0	0.0	16	3.3	0.52	0.97	0.40
Percutaneous exposure (shaving at a barbers shop)	3	25	300	61.5	0.011	0.2	6.52
Tattoo history	3	25	8	1.6	0.000	20	29.7
Piercing	1	8.3	5	1	0.02	20	5.27
Not identified	0	0.0	13	2.7	0.56	0.97	0.32

OR, odds ratio.

Significant relationship between viral hepatitis delta and some of these factors investigated was found, such as number of children (OR = 5.6, *p* = 0.001) and obesity (OR = 4.19, *p* = 0.000) (Table 1).

Diabetes (OR = 6.83, *p* = 0.000), ALT (OR = 6.9, *p* = 0.005) and HVB DNA (OR = 5.69, *p* = 0.003) were correlated with anti-HDV serology antibodies (positive or negative) (Table 1).

Prevalence of HDV seropositivity varies according to the liver disease stages: acute hepatitis (3.6%) with a 95% CI 0% – 9%, chronic hepatitis (1%) with a 95% CI 0% – 2%, cirrhosis (11.1%) with a 95% CI 1.8% – 20.3% and hepatocellular carcinoma (0%) (Table 4).

**TABLE 4:** Prevalence of hepatitis delta virus infection among hepatitis B virus (HBV) infected subjects with liver disease.

HBV-related liver disease group	*n*	IgG-anti-delta positivity		*P*	95% CI	0R
		Positive	%			
Acute hepatitis	55	2	3.6	0.52	0–9	-
Chronic hepatitis	384	4	1	0.000	0–2	0.2
Cirrhosis	54	6	11.1	0.000	1.8–20.3	9.6
Hepatocellular carcinoma	7	0	0.0	0.67	-	-
**Total**	**500**	**12**	-	**-**	**-**	**-**

OR, odds ratio.

## Discussion

This study demonstrates that Algeria is in a low endemic region for VHD. The available data studies on hepatitis delta are exposed in Table 5.^18,19^

**TABLE 5:** Prevalence of viral hepatitis delta in Algeria.

Study	Acute hepatitis		Chronic hepatitis		Cirrhosis		Hepatocellular carcinoma		Prevalence	
	%	Positive (*n*)	%	Positive (*n*)	%	Positive (*n*)	%	Positive (*n*)	%	Positive (*n*)
Nouasria 1984	6	3/50	-	-	-	-	-	-	6	3/50
Belabbes 1986	3.7	3/81	16.6	1/16	15.1	5/33	-	-	7.5	9/120
Berkane 2003	-	-	6.81	3/44	-	-	-	-	6.81	3/44
Khelifa 2009	-	-	1.33	1/75	-	-	-	-	1.33	1/75
Our study	3.6	2/53	1	4/384	11.1	6/54	0	0/7	2.4	12/500

Our prevalence is comparable to those found in most of the Maghreb countries (Tunisia: 6.8%,^20^ Egypt: 4.7%,^21^ Morocco: 1.17%,^22^ and Libya: 10.8%^23^).

**FIGURE 2:**
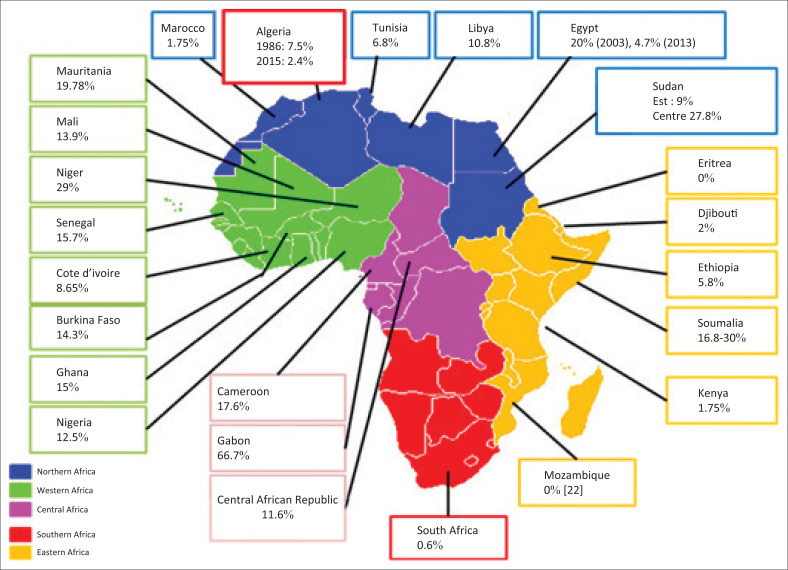
Prevalence of hepatitis delta in Africa.

In contrast to our results, Mauritania shows a high prevalence of 19.7%,^15^ and in Central Africa, Makuwa^24^ has reported a very high prevalence of 66.7% in Gabon, recalling the first outbreaks of hepatitis delta described in the 1980s in the Central African Republic.^25^ In most countries of West Africa,^26^ especially those sharing borders with Algeria, high rates are recorded. Our prevalence is above that reported by Dusheiko in South Africa^27^ (0.6%) (Figure 2).

Our results are lower than the higher rates reported in Italy; as in several European countries, Italy has witnessed a significant decrease in its VHD infection rates.^12,28^

This study allowed us to have updated information about the infection in at least three cities. The prevalence varies from one city to another. This difference in prevalence between the three cities may be related to the incidence of viral hepatitis B (estimated in Algeria at 2.16%, and 2.68% in Msila city).^29,30^ This same observation was reported by Djebbi^20^ in Tunisia.

The prevalence of hepatitis D (HVD) in Algeria is closely related to gender and age. In most studies, a male predominance has been noted^8,29^ with the exception of some countries or some regions. More recently, the Hepatitis Delta International Network (HDIN) Register^31^ which includes 12 worldwide study centres for delta hepatitis, reported results showing predominance of one gender over the other according to the patient origin.

This study has provided us with some information on the exposure of risk factors in life. The presence of the family cases of viral hepatitis B is one of the found risk factors which is in agreement with that reported by Fattovich^32^ in Italy (8.1%, *p* = 0.004).

The second risk factor found was the endoscopic procedure. Few studies have focused on this mode of transmission. Our results are in agreement with those of Gheorghe in Romania^33^ (36.8%, *p* = 0.0001).

The risk of virus transmission during endoscopy is low, because the cleaning and disinfection practice insures a significant viral inactivation.^34^

Thirdly, tattooing was found to be a significant risk factor in patients with VHD.

During tattooing, the muco-cutaneous barrier is broken accompanied by a break of blood capillaries, leading a moderate and transient bleeding which is enough exposure to the risk of infections with hepatitis viruses or HIV.

Traditional methods of tattooing and poor hygiene practices contribute mainly to the increased risk of transmission. Exposure to other risk factors is not significant.

This is the only study that was interested in the association of delta hepatitis and diabetes. The diabetic population is being exposed to a multiplicity of risk factors for hepatitis; furthermore, diabetes favours the development of severe forms of liver disease (cirrhosis and hepatocellular carcinoma).

Our study sheds light on the prevalence of hepatitis delta virus at different stages of liver disease. This rate is consistent with that found by Belabbes^18^ who has reported a prevalence of 15.15% (5/33). Several authors have noted the frequency of anti-HDV antibodies in patients at the cirrhosis stage.^35^

In conclusion, our results show that HDV infection is low in Algeria.

Research for total anti-HDV should be part of the initial assessment of patient care with viral hepatitis B as well as the search of other viruses (HCV and HIV), and the completion of a multicentre study should be carried out to establish the prevalence of HDV and identify the risk groups.
